# Integrated Interventions to Tackle Antimicrobial Usage in Animal Production Systems: The ViParc Project in Vietnam

**DOI:** 10.3389/fmicb.2017.01062

**Published:** 2017-06-13

**Authors:** Juan J. Carrique-Mas, Jonathan Rushton

**Affiliations:** ^1^Oxford University Clinical Research Unit, Hospital of Tropical DiseasesHo Chi Minh City, Vietnam; ^2^Institute of Infection and Global Health, Faculty of Health and Life Sciences, University of LiverpoolLiverpool, United Kingdom

**Keywords:** antimicrobial usage, Vietnam, interventions, poultry, veterinary medicine

## Abstract

Antimicrobial usage and antimicrobial resistance (AMR) in animal production is now recognized to be an important contributor to the global problem of AMR. Initiatives to curb indiscriminate antimicrobial use in animal production are currently being discussed in many low- and middle-income countries. Well-designed, scientifically sound interventions aimed to tackle excessive antimicrobial usage should provide scientists and policy makers with evidence of the highest quality to guide changes in policy and to formulate better targeted research initiatives. However, since large-scale interventions are costly, they require careful planning in order not to waste valuable resources. Here, we describe the components of the ViParc project (www.viparc.org), one of the first large-scale interventions of its kind to tackle excessive antimicrobial usage in Southeast Asian animal production systems. The project has been formulated as a “randomized before-and-after controlled study” targeting small-scale poultry farms in the Mekong Delta region of Vietnam. It aims to provide farmers with a locally-adapted veterinary support service to help them reduce their reliance on antimicrobials. ViParc has been developed in the backdrop of efforts by the Government of Vietnam to develop a National Action Plan to reduce Antimicrobials in Livestock and Aquaculture. Crucially, the project integrates socio-economic analyses that will provide insights into the drivers of antimicrobial usage, as well as an assessment of the cost-effectiveness of the proposed intervention. Information generated from ViParc should help the Government of Vietnam refine its policies to curb excessive antimicrobial usage in poultry production, while lessons from ViParc will help tackle excessive antimicrobial usage in other productions systems in Vietnam and in the broader Southeast Asian region.

## Background

Antimicrobial resistance (AMR) in food animal production systems has recently become the focus of considerable scientific attention. There is now a consensus that AMR represents a “One Health”/“Ecohealth” challenge that urgently needs to be tackled by the international community. A number of high-level reports have indicated the urgency to formulate and conduct interventions to curb antimicrobial usage and AMR, both in human medicine as well as in animal production. Potential interventions may involve education campaigns, improved awareness and farmer training, the provision of antimicrobial replacements, changes in the antimicrobial supply system, or changes in policy and legislation (Anon, 2016a,b).

Initiatives to curb antimicrobial usage and to monitor AMR in food animal production systems have been on-going for over a decade in many developed countries, and in some cases (i.e., Scandinavian countries) for even longer. However, in developing countries such initiatives are still embryonic. This is quickly changing, and momentum is building through the support of international organizations and donors such as FAO, OIE, European Union, Wellcome Trust, Bill & Melinda Gates Foundation, the Fleming Fund, etc., This interest is providing impetus and funding for researchers to develop and implement scientific approaches that help fill existing knowledge gaps on antimicrobial usage and AMR, to design interventions, and to assist policy-making at all levels.

However, examples of scientifically sound large-scale interventions to curb antimicrobial usage in animal production are still rare. Given the complexity and diversity of animal production systems worldwide, their variable antimicrobial usage practices, the baseline levels of AMR, as well as the cultural and legal frameworks in which they operate, it is unlikely that “one-size interventions” would fit all. In order to be meaningful for policy makers and producers, well-designed interventions need to reflect such complexity and diversity. Typically intervention studies are costly since they require a considerably large number of study units (farms, geographical units) to be truly representative, and to be carried out over a long period (no less than a year). Maintaining producer interest over such a long period is likely to represent an additional challenge. With these considerations, careful design and planning is crucial in order to optimize limited resources. On the benefit side, well-planned and conducted interventions may produce results that may be of benefit to sectors beyond the specific target production system. A crucial step is the development of adequate tools and processes that meet the criteria of feasibility, sensitivity, relevance, flexibility, and cost-effectiveness within a specific context (Smith et al., 2001).

In Vietnam, antimicrobials destined for food animal production are managed by the Government through the Ministry of Agriculture (MoA), although the Ministry of Industry and Trade has also some competences. The MoA regularly publishes and updates a list of approved as well as a list of banned veterinary drugs and antimicrobial growth promoters following the submission of technical dossiers by commercial companies. Currently there are ~2,000 veterinary drugs containing antimicrobial ingredients currently licensed for sale in Vietnam. A number of antimicrobials have added to the banned list over recent years, but the current approved list includes a large number of antimicrobials of families that are currently classified as of critical importance by WHO. Once the supplier has obtained official government approval, there are no further sale restrictions, and countries are not require to report on sales of antimicrobials. Most antimicrobial sales are done through veterinary pharmacies, which are licensed by the local veterinary authority. Purchasing of antimicrobial drugs for animal use require no veterinary prescription. There is an on-going Government-funded programme to monitor residues in meat, fish, and shrimp, as well as the quality of the antimicrobial and feed supply chain.

A considerable body of evidence has been accumulating on levels of current antimicrobial usage in poultry production systems in Vietnam. This includes high levels of usage of antimicrobials as growth promoters (Van Cuong et al., 2016), but also as prophylactic and therapeutic agents (Carrique-Mas et al., 2014; Nhung et al., 2014). The reasons for this pattern of antimicrobial use are complex but it is hypothesized that in addition to a lax procurement system, is also partly related to the high incidence of infectious diseases, and crucially a lack of veterinary professionals that can provide unbiased advice to farmers, other than pharmacists and commercial companies with a vested interest in selling antimicrobials.

## The ViParc project

**Figure 1:**
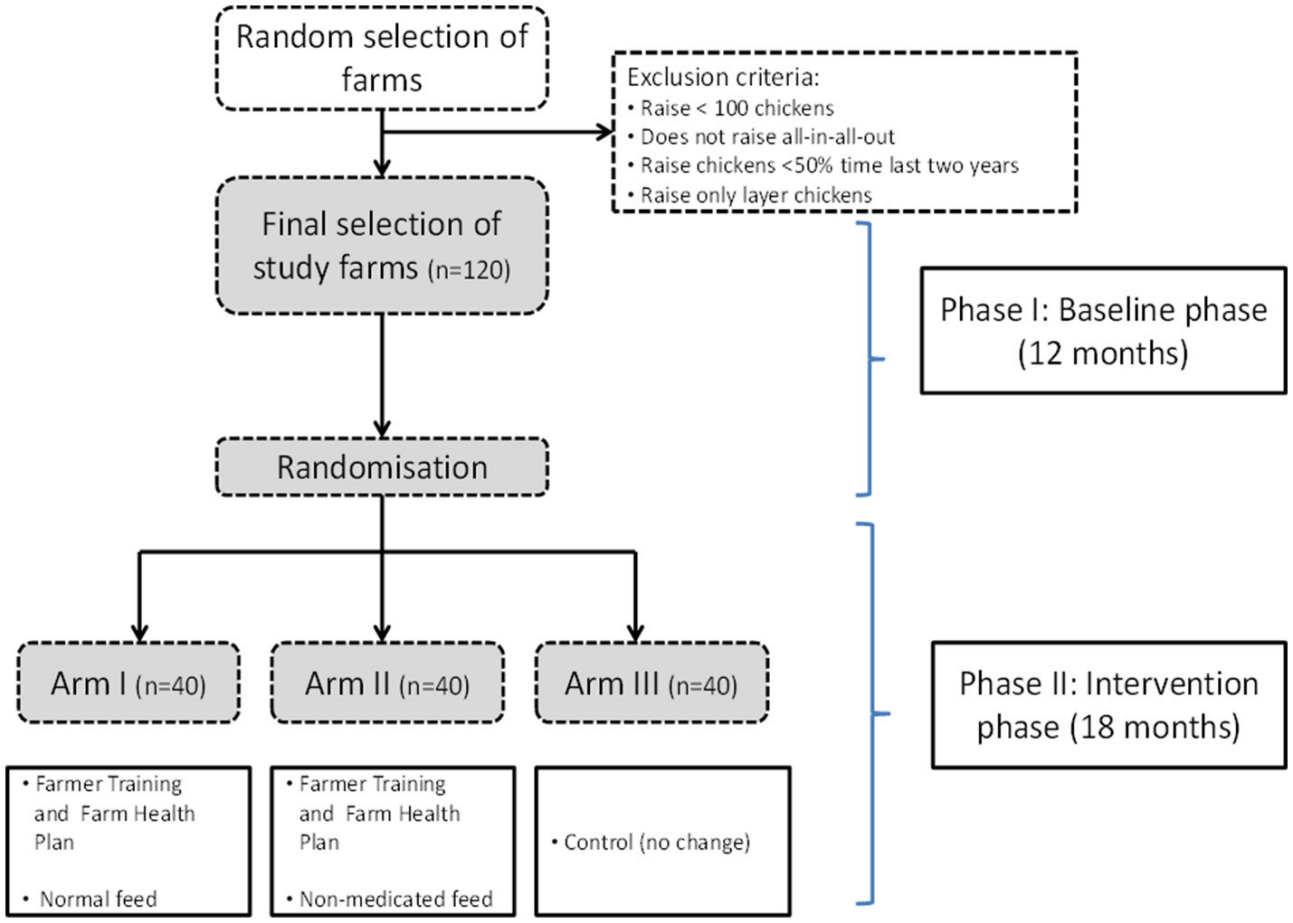
Study design of the ViParc intervention study.

The ViParc project (acronym for “Vietnamese Platform for Antimicrobial Reduction in Chicken production”; http://www.viparc.org), funded by the Wellcome Trust, is one of the first scientifically-based intervention trials of its kind aiming to tackle antimicrobial usage in food animal systems in the Southeast Asian region. The project targets the small poultry sector which is currently prevalent in Vietnam. It involves the recruitment and follow-up of 120 randomly selected meat chicken farms from the Mekong Delta. The study has been designed as a “randomized before-and-after controlled trial” in order to achieve the greatest level of scientific evidence. The project will be delivered in two phases, a “baseline” phase (12 months), followed by an intervention phase (18 months). The “intervention” will consists of the provision to randomly selected farmers in the intervention group with a cost-free, locally-adapted advisory system backed by diagnostics to identify the most common diseases affecting poultry in the area (See later). A control arm will not receive any veterinary support. Because about 50% of the chicken feeds are medicated (Van Cuong et al., 2016), one intervention arm will also have compulsory replacement of medicated feed with non-medicated withdrawal of. The trial design is shown in Figure 1.

The ViParc project will scientifically test the hypothesis that the lack of unbiased poultry veterinary advisors contributes to the practice of farmers buying antimicrobials “over the counter” to avert the risk of disease on their farms. With the help of international experts, ViParc is currently developing capacity of a small cadre of ViParc “poultry vets” that will be responsible for delivering the intervention to farms allocated to the intervention group. The research design will answer if antimicrobial usage in the intervention vs. the control group (no intervention) is different and the prevalence of antimicrobial resistant bacteria and residues in meat before and after the intervention. Data from the project will also capture whether the productivity and profitability of the poultry systems is altered by changes in veterinary advice, antimicrobial usage, and potentially AMR levels.

Crucially, the ViParc project is concerned with the collection of high quality data. This has been a challenge in many previous projects that involved unannounced visits to farms. This is due to the fact that in most cases smallholder farmers do not keep adequate records, and the farmer typically has little recollection of previous practices. In addition, there is often a certain mistrust often precludes the provision of accurate data to the interviewers. The ViParc project involves repeated visits to the farms (three per cycle) by well-trained animal health workers. Farmers are required to keep a project log-book where all relevant data (antimicrobial and feed consumption, disease, mortality, vaccination, etc.) is weekly annotated. Farmers are also asked to keep containers of all medicines and commercial feed products used. An economic report will be generated from the data on real time. A summary of this will be fed-back to the farmer to maintain their interest and commitment to the project. In addition, fecal samples are collected from the flocks at each production phase to investigate AMR in commensal enteric flora. This information will be used to describe changes in prevalence of resistance over time and elucidate the relationship between antimicrobial usage and AMR. Finally, chicken meat is investigated for residues at the end of each production cycle.

## The vietnamese policy context

The ViParc project takes place in a context of high-level activity aiming to curb and monitor AMR in animal productions systems in Vietnam. With the support of the Food and Agriculture Organization of the United Nations (FAO), the Ministry of Agriculture of Vietnam is currently developing its “National Action Plan for the Reduction of Antimicrobial Use and Antimicrobial Resistance in Livestock Production and Aquaculture.” The plan has been structured around four specific objectives: (1) to revise and enforce policy and governance related to AMR and AMU in livestock production and aquaculture; (2) to increase awareness on AMU and the risk of AMR occurrence among livestock and aquaculture professionals, producers and consumers; (3) to promote responsible AMU and husbandry practices among livestock and aquaculture producers; and (4) to monitor AMR, AMU, and antimicrobial residues in livestock production and aquaculture. It is expected that the ViParc project will contribute to outputs within objectives (1), (2), and (3). Given the commitment of the Government of Vietnam, it is highly likely that during the implementation of ViParc restrictions will be implemented related to the availability of certain antimicrobials (i.e., growth promoters). The continued monitoring of “control farms” as part as the ViParc project, will allow to measure the impact of some of these changes.

## Components of the ViParc intervention

An intervention “package,” provided free of charge to farms randomized to the two intervention arms, will consist of the provision of a locally-adapted veterinary support system. The components of this support system are:

1. *A Farmer Training (FT) module*. The FT module consisting of three workshops where farmers will be trained by poultry specialists on: (i) Good farming practices: record keeping, chick procurement, water and feed quality, farm biosecurity, cleaning and disinfection, and pest control; (ii) Control of poultry diseases, including responsible use of antimicrobials, vaccines, and probiotics; and (iii) Waste management and environmentally-sustainable practices.
2. *A Farm Health Plan (FHP)*. Each farm will be allocated to one poultry vet at the beginning of the intervention who will draft a tailor-made Farm Health Plan (FHP). The FHP document will include specific recommendations to improve the flocks' health, likely to include advice on vaccination schedules, detailed plans for terminal C&D and improvements in biosecurity. The poultry vet will visit each farm once during each production cycle to inspect the flocks, review records, and update the FHP document. In addition, and with the help of international experts, the project is developing and Auditing Tool in order to score each farm on a number of key variables, and to measure potential take up of the advice given by the poultry vets.
3. *A Diagnostic Support programme*. The ViParc project will set up a basic diagnostic laboratory that will cover the most common poultry bacterial, viral and parasitic diseases. Farmers in the intervention arm will be instructed to alert their project vet should they observe signs of disease in their flocks. Poultry vets will carry out cost-free diagnostic investigations aimed at establishing the cause of disease, focused on identifying the most common bacterial, parasitic, and viral diseases known to be present in the area. The information generated during the project will be used to improve the advice given on vaccination in the area. However, setting up diagnostics is costly and complex. It is envisaged that the project will focus only on the most common bacterial and parasitic diseases circulating in the area. The diagnostic methods that will become available to the laboratory are likely to include bacterial culture for bacterial pathogens, testing of viral pathogens (by conventional PCR), and quantification of helminths. Bacterial pathogens isolated from diagnostic specimens will be investigated for their antimicrobial susceptibility against commonly used antimicrobials. Although, it is unlikely that this may influence treatment of specific disease incidents, since farmers will normally not wait until laboratory confirmation. However, this will allow build up a basis of knowledge on AMR among poultry bacterial pathogens in the area, which will be extremely valuable to inform treatment guidelines.

An important consideration is that the ViParc intervention will follow a “persuasive” rather than a “restrictive” approach, with no formal enforcement of restrictions to the farmer on antimicrobial usage. It is expected that through on-going advice a considerable amount of disease will be averted on farms, and it is expected that positive changes in productivity will be detectable in the intervention group.

## Behavioral studies and socioeconomic analyses

Since poultry farms, however small, operate on a business rationale, the use of antimicrobials, and other health-enhancement practices are likely to respond to economic drivers. However, these behaviors are often based on existing perceptions of risk and cultural attitudes. Because of this, a component of the ViParc project includes the investigation of the socio-economic context. An essential pre-requisite for the project is the understanding of the supply chain context (i.e., supply of day-olds, feed, antimicrobials, disinfectants, and other health-enhancing products) it will be essential to measure these costs in relation to the benefits (finished chickens, feathers, and manure) and its market fluctuations.

Long term sustainable AMU-reducing interventions require the integration of solid economic data with in-depth knowledge of farmers' beliefs and motivating factors for antimicrobial usage. However, little is known about these in the Vietnamese context. From an economic perspective, a rational farmer would balance the cost of antimicrobials against the avoidance of losses due to disease. However, non-economic factors such as the desire to maintain a particular self-image or social identity, the compliance with social or institutional rules, and cognitive biases in the perception of risk and uncertainty can all influence the perceived cost-benefit balance. Farmers may seek to mitigate this uncertainty by seeking information from sources they believe are better informed (i.e., veterinary pharmacists) that have a vested interest in the sale of antimicrobials.

An economic assessment based on the estimate of the gross margin (output value less variable costs) of the batches of chickens raised will be performed with data generated from the ViParc project. The marginal costs and benefits will be examined using a partial budget analysis framework before and after the intervention. The data to feed these analyses will include: (1) potential changes in costs due to disease and productivity; (2) reduced costs in antimicrobials; (3) costs of the intervention; (4) potential for behavior change; and (5) potential AMR reductions. Crucially the ViParc project aims to understand to what extent the proposed intervention (advice, diagnostic support) is taken up by the farmer. This will be measured as far as possible using several tools, since it is a crucial variable in terms of the formulation of a potential cost-effective package.

## Limitations of the ViParc project

A potential limitation of ViParc is its focus on “full time” dedicated farmers. A large fraction of farmers in the area raise “backyard” flocks with <100 birds, and these farmers are not eligible for the study. In addition, an unknown percentage of poultry farmers only raise chickens depending on the economic and market conditions, and therefore may not be in the official farm census. However, we believe that these farmers are very much unlikely to request veterinary support, and simply cull their birds whenever there is disease in their flocks and move on to another business. The project is implemented through the official provincial veterinary system, since there are no private poultry advisors other than commercial companies and veterinary pharmacists in the area. The level of trust between farmers and the veterinary system at present is quite low, although we expect to see an improvement in this relationship as a result of the ViParc project.

## Research to advice policy

The ViParc project is expected to provide evidence to the Government of Vietnam on whether the proposed intervention will have a behavioral and economic impact, both at farm-level and across the food production chain. Analyses from ViParc will allow the formulation of pricing mechanisms and those components within the intervention package with the highest likelihood of being sustainable in the long term in the Vietnamese context. The development of a cadre of well-trained poultry advisors and an upgrade of existing diagnostic systems are likely outcomes from the project. Where the interventions are not economically profitable at farm-level, yet generate positive externalities in terms of reducing AMR levels on farms, it will be possible to propose modifications for the antimicrobials and chicken meat market. Alongside the formal testing of interventions, the ViParc project will develop of methods and metrics for the assessment in the smallholder farm context, and will shed light on the need for upgrading of existing production systems.

## Ethics statement

The ViParc project has been granted ethics approval by the Oxford Tropical Research Ethics Committee (OXTREC) (Ref. 5121/16).

## Author contributions

JC wrote the initial draft and conceived the idea of the intervention study. JR contributed to write the socio-economics and policy components.

### Conflict of interest statement

The authors declare that the research was conducted in the absence of any commercial or financial relationships that could be construed as a potential conflict of interest.
